# UNDERSTANDING INCLUSIVE PHYSICAL PLACES AND VIRTUAL SPACES: THE FORTITUDE OF OLDER MARGINALIZED COMMUNITIES

**DOI:** 10.1093/geroni/igae098.0022

**Published:** 2024-12-31

**Authors:** Mei Lan Fang, Judith Sixsmith, Susan Buell, Darren Chadwick, Kathryn Almack, Richard Vytniorgu, Joe Tai

**Affiliations:** Simon Fraser University, Vancouver, British Columbia, Canada; University of Dundee, Dundee, Scotland, United Kingdom; University of Dundee, Dundee, Scotland, United Kingdom; Liverpool John Moores University, Dundee, Scotland, United Kingdom; University of Hertsfordshire, Hertfordshire, Scotland, United Kingdom; University of Hertsfordshire, Hertfordshire, England, United Kingdom; University of Dundee, University of Dundee, Scotland, United Kingdom

## Abstract

The World Health Organization (WHO) Age-Friendly Cities and Communities (AFCC) agenda emphasizes collaboration among older individuals, local groups, councils, and businesses to enhance communities. This involves improvements in several social and environmental domains including transportation, outdoor spaces, volunteering, employment opportunities, and leisure and community services. IncludeAge, a UK Economic and Social Research Council funded project, addresses multifaceted aspects of age-friendly communities beyond the emphasis on social and physical environments, highlighting the importance of belonging and identity alongside cultural and life course experiences. Focusing on physical and online places and spaces, IncludeAge takes intersectional and life course perspectives to understanding the inclusion of mid-older (40+) people who are often marginalized: those with intellectual disabilities and LGBT+ individuals. Employing a transdisciplinary, innovative mixed-methods approach and guided by Community-Based Participatory Research principles, our longitudinal study uses life course interviews, GIS story mapping and social network analysis to document experiences of inclusion and exclusion in past lives and current real-time. Preliminary insights underscore the importance of the cultural and historical dynamics of experienced stigma and discrimination, the value of ‘hidden spaces and places’ and ‘experiences on the periphery’. We use the Intersectional Place Perspective (IPP) theoretical model to aid interpretation. Through co-developed story maps and individual narratives, we aim to amplify the voices of marginalized groups in designing potential solutions to exclusionary experiences and practices, fostering empowerment and social inclusion. Our research will advance understanding of AFCC and community inclusion as a human right, thereby supporting meaningful everyday lives for our focal populations.

